# Disparate phospho-Smad2 levels in advanced type 2 diabetes patients with diabetic nephropathy and early experimental db/db mouse model

**DOI:** 10.1080/0886022X.2017.1361837

**Published:** 2017-08-14

**Authors:** Lise Høj Thomsen, Morten Fog-Tonnesen, Lisbeth Nielsen Fink, Jenny Norlin, Amaya García de Vinuesa, Troels Krarup Hansen, Emile de Heer, Peter ten Dijke, Alexander Rosendahl

**Affiliations:** a Department of Diabetes Complications Research, Novo Nordisk A/S, Måløv, Denmark;; b Department of Endocrinology and Internal Medicine, Aarhus University Hospital, Aarhus, Denmark;; c Department of Incretin & Obesity Pharmacology, Novo Nordisk A/S, Måløv, Denmark;; d Department of Molecular Cell Biology, Cancer Genomics Centre Netherlands, Leiden University Medical Center, Leiden, The Netherlands;; e Department of Pathology, Leiden University Medical Center, Leiden, The Netherlands

**Keywords:** Type 2 diabetes, diabetic nephropathy, fibrosis, TGF-β, BMP, kidney

## Abstract

Uncontrolled activation of transforming growth factor beta (TGF-β) family members is hypothesized to participate in type 2 diabetes (T2D) dependent diabetic nephropathy (DN). We evaluated and compared downstream activation of the Smad2-signaling pathway in kidney samples from T2D patients to kidneys from the T2D model of leptin receptor deficient db/db mouse. Furthermore, expression of TGF-β family members was evaluated to elucidate molecular mechanisms in the mouse model. Kidney samples from patients with advanced stages of DN showed elevated pSmad2 staining whereas db/db mouse kidneys surprisingly showed a decrease in pSmad2 in the tubular compartment. Structurally, kidney tissue showed dilated tubules and expanded glomeruli, but no clear fibrotic pattern was found in the diabetic mice. Selective TGF-β family members were up-regulated at the mRNA level. Antagonists of bone morphogenetic protein (BMP) ligands, such as Gremlin1, USAG1 and Sclerostin, were strongly up-regulated suggesting a dampening effect on BMP pathways. Together, these results indicate a lack of translation from T2D patient kidneys to the db/db model with regards to Smad signaling pathway. It is plausible that a strong up-regulation of BMP antagonizing factors account for the lack of Smad1/5/8 activation, in spite of increased expression of several BMP members.

## Introduction

Metabolic diseases such as obesity and type 2 diabetes (T2D) are closely linked, and 35% of the population is estimated overweight or obese in the Western world, a number which continues to increase [1]. Obesity and T2D are associated with a higher risk of cardiovascular disease (CVD), decreased insulin sensitivity and chronic kidney disease (CKD), which increases mortality beyond the traditional CVD risk factors [2,3]. Obesity-related renal disease is similar to the microvascular damage observed in diabetic nephropathy (DN) [4]. It is believed that kidney damage is a consequence of a combination of increased glomerular capillary pressure and hyperfiltration, endothelial cell proliferation and dysfunction, increased vascular permeability, increased protein traffic, mesangial hyperplasia and renal hypertrophy [5,6]. T2D accounts for more than 90% of the worldwide diabetes incidence, but DN in T2D patients is less well described than T1D patients [7]. T2D patients show larger variation in disease pattern and pathology, which complicates the study of DN within T2D as estimates for rate of disease progression are imprecise and sparse [8–10].

DN is the leading cause of CKD where a hallmark is the development of kidney fibrosis. The Transforming Growth Factor beta (TGF-β) family has been implicated in kidney fibrogenesis evidenced by elevated TGF-β isoforms and increased Smad signaling. These findings are also seen in obese patients and animal models [11–13]. However, the specific role of Smad2 in DN remains unknown.

Patients with DN show increased TGF-β1 mRNA and protein levels in the glomerular and tubulointerstitial compartments [14,15]. Additionally, *in vitro* cell culture experiments demonstrate that hyperglycemia stimulates TGF-β1 production from glomerular and tubulointerstitially derived cells [16–21].

Late stages of DN show glomerulosclerosis with Kimmelstein–Wilson nodules, tubulointerstial fibrosis, thickening of the basement membrane, albuminuria and decline of renal function [22], but no current experimental model is able to fully recapitulate these renal changes. The db/db mouse model shows significant obesity early in life, followed by mesangial expansion, thickening of the glomerular basement membrane (GBM) and proteinuria [23,24]. The model shows hyperlipidemia, obesity, albuminuria, mesangial matrix expansion and immune compromisation [25–27]. Albuminuria is seen at 10–12 weeks, and by week 20, the mice show overt proteinuria and advanced kidney structural damage [25]. Development of diabetes is more severe in the C57BL/KsJ mice compared to the C57BL/6 J mice [28], which is the reason for us using the KsJ strain.

In the current study, we evaluated the similarities and differences in renal compartmental phospho-Smad2 levels between human T2D associated DN and T2D DN mouse model (db/db) to determine how well the disease translates between the species. We further explored mouse TGF-β family expression and activity of the TGF-β counterbalancing Smad1/5/8 pathway to give a mechanistic insight in to the observed species differences. Our data suggest a clear lack of translation between patients and mouse model with regards to TGF-β family induced Smad signaling.

## Methods

### Animal models

Male db/db mice (Strain C57BL/KsJ from Charles River, Germany) were purchased at 6–8 weeks of age and kept according to standard rules at the Novo Nordisk A/S animal facility (Måløv, Denmark). All experiments were approved by the Danish Ethical Committee. Blood glucose was measured by tail vein blood puncture and diabetes defined as three consecutive morning blood glucose readings of >16 mmol/L using Glucose Analyzer Biosen 5040 (Germany). HbA1c% (percentage glycated haemoglobulin) was measured using Cobas 6000 (Switzerland). Albumin in the urine was measured by an enzyme-linked immunosorbent assay (ELISA) kit from Bethyl Labs (USA).

At 15 and 23 weeks of age, the mice were anesthetized, sacrificed and cardiac perfused before the kidneys were isolated for histology and gene expression analysis.

### Histology and immunostaining

Human kidneys were obtained from deceased patients from autopsy material at the Pathology Department at Leiden University Medical Center and stained at this facility. Cause of death was examined by regular pathology. Some kidney samples were obtained from patients that had been selected for organ transplantation. If the patients were eligible for transplantation their organs were prepared by post mortem perfusion of the whole body intravenously with University of Wisconsin fluid. The body was cooled and biopsies of the kidneys were taken for pre-transplant histological analysis. If their kidneys were unsuitable for transplantation because either the ureter or the renal artery were too short, these kidneys were made available for research as control kidneys. If DN was found in donor kidneys by histology, this also disqualified the kidneys for transplantation and these were also made available for research. Clinical symptoms for T2D with DN included polyuria, thirst, hypertension, blurred vision, albuminuria and increased level of serum creatinine according to UK National Health Service. The human experiments are all in agreement with the Declaration of Helsinki and samples were handled anonymously in accordance with the Dutch National Ethics Guidelines (Code for Proper Secondary Use of Human Tissue, Dutch Federation of Medical Scientific Societies) meaning that experiments were approved by the institutional ethics board of the LUMC. Kidneys were classified positive for DN by the histopathological classification [29]. The T2D patients in this study only showed DN and not prevalence of other non-diabetic renal disease. All T2D patients had been on angiotensin-converting enzyme inhibitors, which is standard treatment for T2D in the Netherlands. Glomerular filtration rate (GFR) stage was classified by the Kidney Disease Improving Global Outcomes work groups KDIGO [30]. Control kidneys were processed similar to the diabetic kidneys and mouse kidneys by fixation in buffered formalin, processing, paraffin embedment and cut at 4-µm. Antigen-retrieval was done by citric acid buffer (0.01 M, pH 6.0) at 100 °C in a microwave oven for 10 min. Slides were cooled at room temperature (RT) for 20 min. Sections for immunostaining were incubated for 20 min with 0.3% H_2_O_2_ in absolute methanol to quench endogenous peroxidase activity. Blocking took place at RT using phosphate buffered saline (PBS) containing 0.1% BSA and 5% goat serum (Dako). Human and mouse slides were incubated overnight at 4 °C with primary polyclonal rabbit antisera pSmad2 diluted in PBS with 1% BSA in a dilution of 1:100 (a concentration of 14ug/ml total protein) (described in [31–33]). This antibody is specific to phosphorylated Smad2 and does not cross-react to other phosphorylated Smads on immunoprecipitation [33]. Mouse slides were also incubated with primary polyclonal rabbit antibody pSmad1/5/8 (#9511, Cell Signaling) diluted in PBS with 1% BSA in a 1:100 dilution (concentration of 14 ug/mL) overnight at 4 °C. Normal rabbit serum diluted to the same concentration worked as negative control. After 3x5-min washes in PBS, the slides were incubated for 30 min with HRP-conjugated anti-rabbit EnVision (#K4002, Dako) at RT. After 3x5-min washes in PBS the antibody complexes were visualized using EnVision + System-HRP (DAB) (#K4011, Dako). Slides were counterstained with hematoxylin, then dehydrated and mounted. The degree of anti-pSmad immunostaining was scored independently by two individuals in a blinded fashion and separately in areas of glomeruli, proximal tubular cells (PTECs) and distal tubular cells (DTECs). For the mouse tissue, the expression within the glomeruli was quantified by percent positive nuclei/total nuclei in 20 glomeruli per kidney. Degree of staining of both PTECs and DTECs was done by grading the degree of positive nuclei staining from 0–3 in the whole tissue for each structure. The human tissue was also graded 0–3. It was noted whether staining was in the Bowman’s capsule, nuclear or cytoplasmic.

Sections for analysis of morphological changes were stained for hematoxylin and eosin (H&E), Periodic Acid-Schiff (PAS) and Picrosirius red. Morphometric analysis of glomeruli and tubular lumen size was measured manually in Phillips viewer program. Glomerular area was measured by drawing the circumference around the glomerular capillary tuft (without Bowman’s capsule). An average of 20 glomeruli was examined per mouse. Distal tubulus lumen diameter was examined by measuring the perpendicular line between the basement membranes within the tubular lumen. An average of 70 tubules was measured per mouse.

### Analysis of mRNA

Kidney homogenates in Trizol were made by use of the TissueLyser II system (Qiagen). mRNA was isolated with a RNeasy Mini kit (#74106, Qiagen) and reverse transcribed according to the manufacturer’s instructions. Gene expression analysis by TaqMan Microfluid card with predefined primer sets was performed by quantitative real-time PCR and cycle threshold (Ct) values of the gene targets were normalized to the average of four housekeeping genes (18 S, Ribosomal Protein L27(Rpl27), Ribosomal Protein S13 (Rps13) and Ubiquitin C (UBC)) found unchanged in the kidney. Fold change in expression of target genes compared with the control reference group was calculated using relative quantitation of gene expression by the 2^-DDCT^ method recommended by Applied Biosystems. Qlucore Omics Explorer 3.2 (Qlucore, Lund, Sweden) was used to analyze 44 genes (Ct(target) – Ct(average of ref genes)) and create a heat-map ranking the genes based on *p* values when comparing db/db to db/+. To avoid false positive significance the false discovery rate was set to <.05 corresponding to a *p* values considered significant when <.024.

### AlphaLISA SureFire ultra pSmad1 assay

The mouse PTEC cell line TCMK1 (#CCL-139™, ATCC^®^) was seeded at a density of 10.000 cells/well in 96 well plates for 24 h. Recombinant Gremlin1 (#956-GR, R&D Systems) was pre-incubated with 4 ng/mL BMP-4 (and 150 ng/mL BMP-7, equivalent to EC80) for 15 min. This mixture was added to the PTECs for 90 min at 37 °C, 5%CO_2_ and 5%O_2_. Cells were lysed with a 1x lysis buffer from the *SureFire Ultra* kit (#ALSU-PSM1-A500, PerkinElmer) and handled according to the manufacturer’s instructions. Light emission in the sample correlates to the quantity of pSmad1 protein. The plate was read on an EnVision Wallac, PerkinElmer.

### Statistical analysis

Biochemical and structural measurements were compared by unpaired unequal variances *t*-test (Welch’s *t*-test) (two-tailed).

Difference in pSmad immunostaining was tested by an unpaired, nonparametric Mann-Whitney (two-tailed) test. This was chosen as the scoring values do not represent normally distributed data.

The gene expression data are shown as fold changes (Box and whiskers with range). Target genes were compared with the control reference group using the 2^-DDCT^ method. Statistics were calculated using the delta-Ct values for an unpaired unequal variances *t*-test (Welch’s *t*-test) (two-tailed).

For all tests, except gene expression, *p <* .05 was considered significant. Statistical analyses were performed using GraphPad Prism 5 (GraphPad software Inc., San Diego, CA).

## Results

### T2D nephropathy is associated with an increased glomerular and tubular cytoplasmic pSmad2 signature

DN associated fibrosis is considered to involve an imbalance of the TGF-β family activity causing elevated pSmad2 signaling in diabetic patients [12,13]. We performed an in-depth immunohistochemical evaluation of kidneys from DN classified T2D patients to determine how the signaling signature of pSmad2 was altered compared to healthy control kidneys. Diabetic kidneys were classified stage 2B and 3 of DN and had a GFR ≤60 (Table 1). Blood pressure and gender distribution were similar between controls and patients, while the patients showed a higher average age than controls (Table 1).

**Table 1.** Clinical data of T2D patients and controls.

Diabetes vs. controls	Age (years)	Gender	Cause of death	DN classification (1–4)	GFR stage (1–5)	GFR (ml/min)	Creatinine (ml/l urine)	Blood pressure (mmHg)
T2D	85	Female	Accident	3	5	11	206	110/70
T2D	69	Male	–	3	3	47	81	–
T2D	75	Male	Pneumonia	2B	2	60	71	130/60
T2D	88	Female	Shock	2B	4	20	331	120/75
Ctrl 1	41	Male	Aneurysm	–	–	–	184	122/87
Ctrl 2	74	Female	Sub arachnoid bleeding	–	–	–	58	155/65
Ctrl 3	68	Male	Cardiac arrest	–	–	–	85	140/78
Ctrl 4	40	–	Sub arachnoid bleeding	–	–	–	–	–

Clinical data of T2D patients with DN and control subjects. DN histopathological classification by Tervaert et al. 2010 [29]. GFR classification by KDIGO 2012 Clinical Practice Guideline for the Evaluation and Management of Chronic Kidney Disease [30].

**Figure 1.**
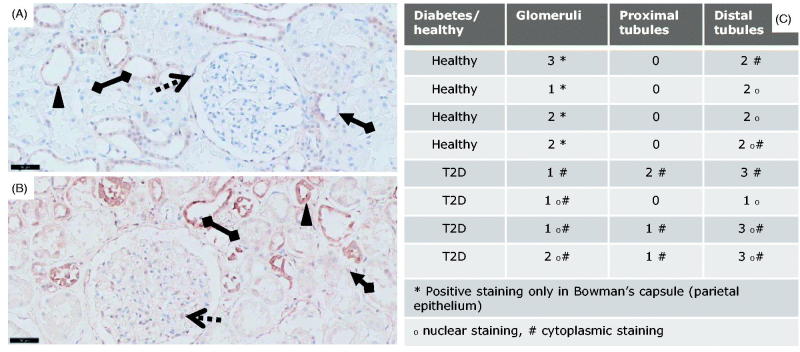
pSmad2 immunostainings of T2D patients vs. controls. pSmad2 immunostaining of kidneys from T2D patients compared to healthy donor kidneys. Representative IHC sections from a healthy donor kidney (A) and a T2D patient kidney (B) shown at 20X magnification where the black-line =50uM. Kidney sections show a glomerulus structure (dotted arrow), a proximal tubule (arrow connected to a diamond) and a distal tubule (diamond connected to diamond). Filled triangle indicates positively stained nuclei structure indicative of active signaling.

Glomerular cells of healthy subjects showed a low nuclear expression of pSmad2 mainly in the Bowman’s capsule (Figure 1(A)): whereas in diabetic patients the glomerular cells were enlarged due to sclerosis and showed an overall elevated cytoplasmic and nuclear pSmad2 staining (Figure 1(B,C)). The expression of pSmad2 within the glomeruli in the T2D patients showed a higher degree of heterogeneity (cytoplasmic and nuclear) compared to what was observed within the glomeruli of the healthy subjects (Figure 1(A–C)). In general, kidneys from the healthy subjects showed clear nuclear staining primarily localized within the parietal cells of the Bowman’s capsule and little cytoplasmic activation, while the DN kidneys showed an overall increased cytoplasmic pSmad2 expression.

The PTECs were pSmad2 negative in the healthy kidneys whereas the DTECs showed a variation from low to moderate nuclear staining of pSmad2 (Figure 1(A,C)). Diabetic tubuli showed a variation in pSmad2 staining in samples from diabetic patients (Figure 1(C)). Importantly, a small increase in PTECs and moderately elevated pSmad2 levels within the DTECs were observed in the DN subjects (Figure 1(B,C)). The elevated pSmad2 expression signature in the epithelial compartment was as in the glomeruli mostly associated with increased cytoplasmic levels and showed a higher individual variation compared to the healthy kidneys.

Taken together, an elevated cytoplasmic pSmad2 signature was present in both the glomerular structures and in the tubular compartment in diabetic kidneys. These results suggest that a disease-related TGF-β-family induced pSmad2 signaling signature is present in T2D DN, but mostly associated with cytoplasmic localization.

### Significantly increased glomeruli size and expanded lumen in distal tubules in db/db diabetic mice with minimal fibrotic signature

The poor translation in pathology from mouse models to patients within progressive renal disease has hindered development of novel treatment regimens [34]. In this study we used db/db mice on a more severe diabetes prone background, the C57BL/KsJ. The diabetic db/db mice showed significantly increased weight and HbA1c compared to db/+ mice (Table 2), while difference in kidney weight was minimal compared to healthy mice. Furthermore, the diabetic animals showed a strong and significant induction of albuminuria (Table 2).

**Table 2.** Characteristics of db/db and db/+ mice.

	db/+ CTRL (*n* = 11)	db/db (*n* = 17)
Weight (g)	32.40 ± 0.48	54.58 ± 1.75
Kidney (g)	0.22 ± 0.01	0.24 ± 0.00
HbA1c (%)	4.29 ± 0.04	8.52 ± 0.29
Albuminuria (µg/24 h)	79.81 ± 20.30	892.2 ± 77.40

Biochemical data on db/db mice vs. control db/+. Animals for IHC were 23 weeks of age. Table show weight, kidney weight, HbA1c and albuminuria. Values are shown as the mean ± SEM for each group. Values are means ± SEM.

**Figure 2.**
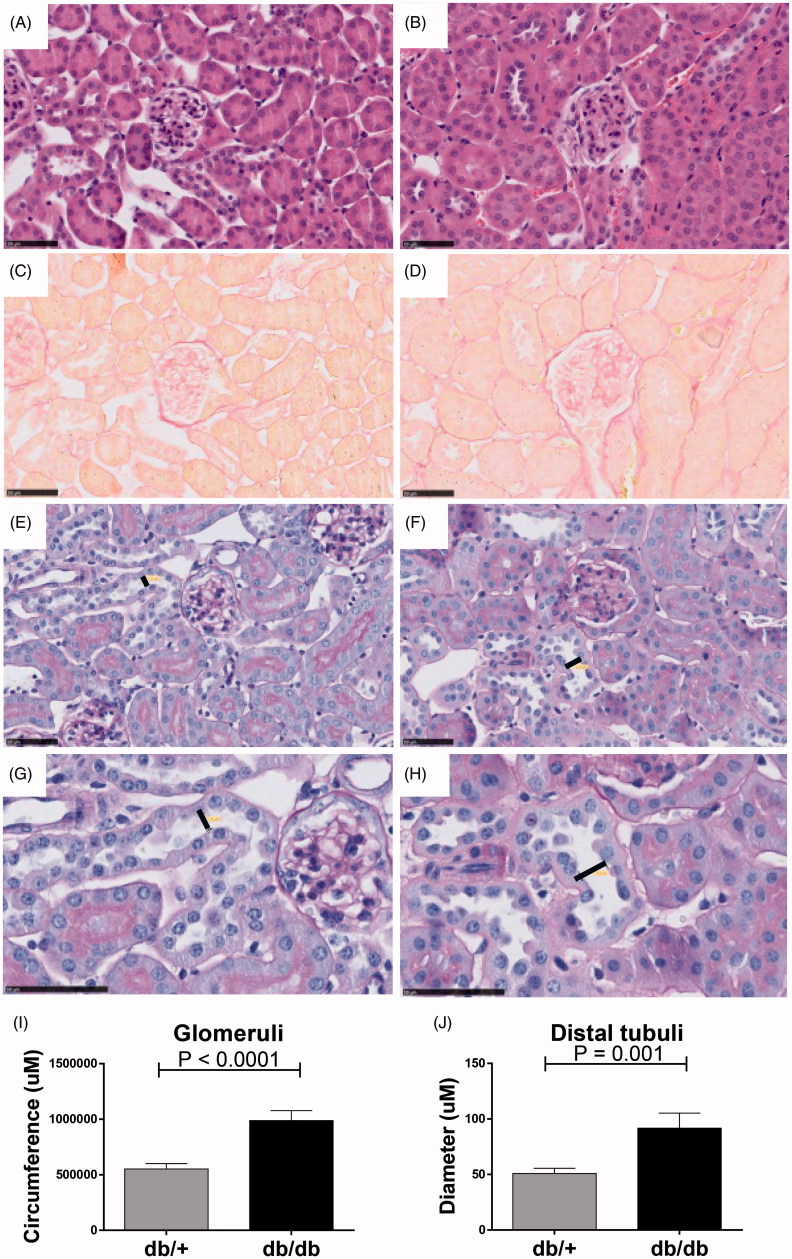
Structural changes in non-diabetic db/+ compared to diabetic db/db kidneys. H&E staining of db/+ (A) and db/db (B) mouse kidney. Picro-sirius red staining of db/+ (C) and db/db (D) mouse kidney. Periodic acid–Schiff staining of db/+ (E) and db/db kidney tissue (F) with magnification of a representative distal tubuli lumen diameter of db/+ (G) and db/db (H) where lumen width is indicated. Quantification of glomerular circumference (I) and distal tubular lumen diameter (J).

Histological evaluation showed that the diabetic mice had structural differences with significantly elevated glomeruli circumference (*p* < .0001) on H&E staining (Figure 2(A,B)) and Periodic acid-Schiff (PAS) staining (Figure 2(E,F,I)). Intriguingly, no fibrotic profile determined by collagen deposits measured by Picrosirius red was observed in the diabetic db/db kidneys either in the glomeruli or in the tubular space (Figure 2(C,D)) at 23 weeks of age. However, the diameter particularly of distal tubuli lumen increased in the db/db mice compared to non-diabetic db/+ controls (*p* = .001) (Figure 2(G,H,J)).

Taken together, the results show that despite having enhanced HbA1c and albuminuria, the db/db mice did not show a collagen-rich fibrotic profile. However, the diabetic db/db kidney showed obvious tissue re-modeling with enlarged glomeruli and distal tubuli lumen present.

### Nuclear pSmad2 translocation is reduced in db/db proximal tubuli cells

Based on the absence of classical fibrotic patterns but clear signs of tissue re-modeling similar to the human DN subjects, the pSmad2 signature was determined by immunohistochemistry in the db/db mice.

**Figure 3.**
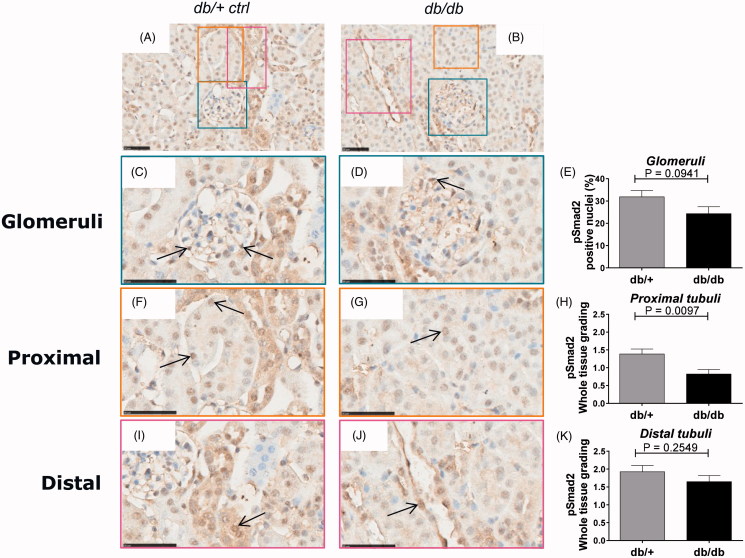
pSmad2 immunostaining in db/+ vs. db/db mice. Representative IHC sections from db/+ control mice (A, C, F, I) and db/db mice (B, D, G, J). Quantification of pSmad2 (E, H, K). (A) and (B) are shown at 40X magnification where the black-line = 50uM. (C) and (D) show a glomeruli structure at 80x magnification, (F) and (G) show a PTEC structure at 80x magnification and (I) and (J) shows a DTEC structure at 80x magnification. Arrows indicate positively stained nuclei structure indicative of active signaling. (E) shows glomeruli quantification. (H) and (K) show PTEC and DTEC quantification. Values shown as means ± SEM.

Overall, both healthy db/+ and diabetic db/db kidneys showed a diffuse staining pattern of pSmad2 throughout the whole kidney (Figure 3(A,B)). Detailed analysis of the glomeruli from db/+ kidneys revealed a moderate expression of pSmad2 with individual cells showing nuclear pSmad2 translocation (Figure 3(C)). Most interestingly, a clear tendency towards an overall decreased pSmad2 expression with reduced frequency of nuclear translocated pSmad2^+^ cells in diabetic glomeruli compared to healthy glomeruli was detected (Figure 3(C–E)).

Healthy PTECs showed a moderate expression of pSmad2 with a number of cells showing nuclear pSmad2 translocation (Figure 3(F)). Similar to the glomeruli, diabetic PTECs showed reduced overall expression and significantly (*p* = .0097) reduced nuclear pSmad2 translocation compared to healthy control mice (Figure 3(F–H)).

DTECs in healthy kidneys showed a moderate expression of pSmad2 with a few cells expressing nuclear translocated pSmad2 (Figure 3(I–K)). The pSmad2 expression signature and level was similar in the diabetic db/db DTEC as in the healthy db/+ DTEC (Figure 3(I–K)).

Taken together, surprisingly both diabetic db/db glomeruli and PTECs showed a decreased expression and nuclear translocation of pSmad2 compared to healthy kidneys suggesting attenuated TGF-β family member signaling in the db/db-T2D kidney.

### No difference in pSmad1 signaling in diabetic kidneys from db/db mice

The pSmad1/5/8 pathway has been shown to counterbalance the TGF-β induced pSmad2 pathway, and is activated by BMPs that are known to participate in kidney homeostasis [35]. In order to determine the pSmad1 signaling signature in kidneys of diabetic db/db and healthy db/+ mice a pSmad1/5/8 immunohistochemiocal analysis was performed.

**Figure 4.**
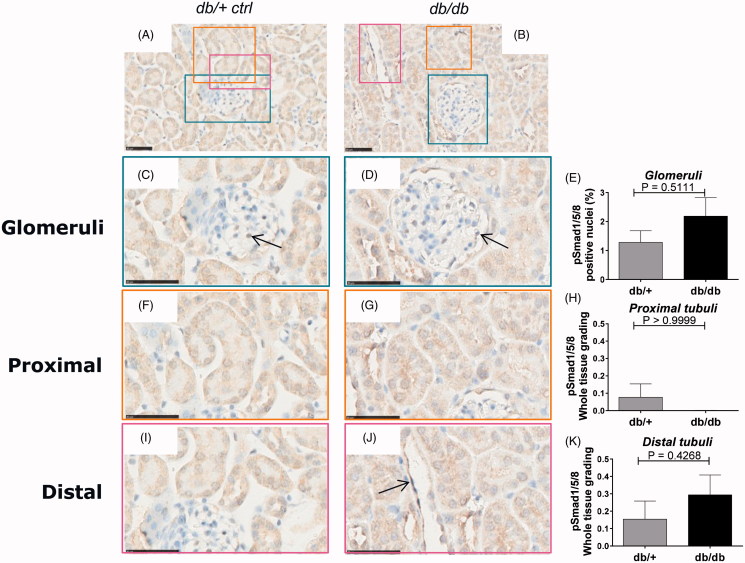
pSmad1/5/8 immunostaining of db/+ vs. db/db mice. Representative IHC sections from db/+ control mice (A, C, F, I) and STZ mice (B, D, G, J). Quantification of pSmad1/5/8 (E, H, K). (A) and (B) are shown at 40X magnification where the black-line = 50uM. (C) and (D) show a glomerulus structure at 80x magnification, (F) and (G) show a PTEC structure at 80x magnification and (I) and (J) show a DTEC structure at 80x magnification. Arrows indicate positively stained nuclear structures indicative of active signaling. (E) shows glomerular pSmad2 quantification. (H) and (K) show PTEC and DTEC quantification. Values are shown as means ± SEM.

A very weak staining with only a few positive stained cells of the glomeruli cells in the non-diabetic kidney was observed. Nuclear translocation in the glomeruli showed no difference between diabetic db/db animals and controls (Figure 4(C–E)). Furthermore, almost undetectable levels of pSmad1 were observed in the PTEC and DTEC compartments in the healthy db/+ kidneys with no difference in nuclear staining between the diabetic and non-diabetic group (Figure 4(F–H) and (I–K), respectively).

### A clear BMP ligand and antagonist signature is present in the diabetic db/db kidney

Based on the altered pSmad signaling signature observed in the diabetic kidneys, the regulation of a number of key TGF-β family members were subsequently analyzed on the gene level.

**Figure 5.**
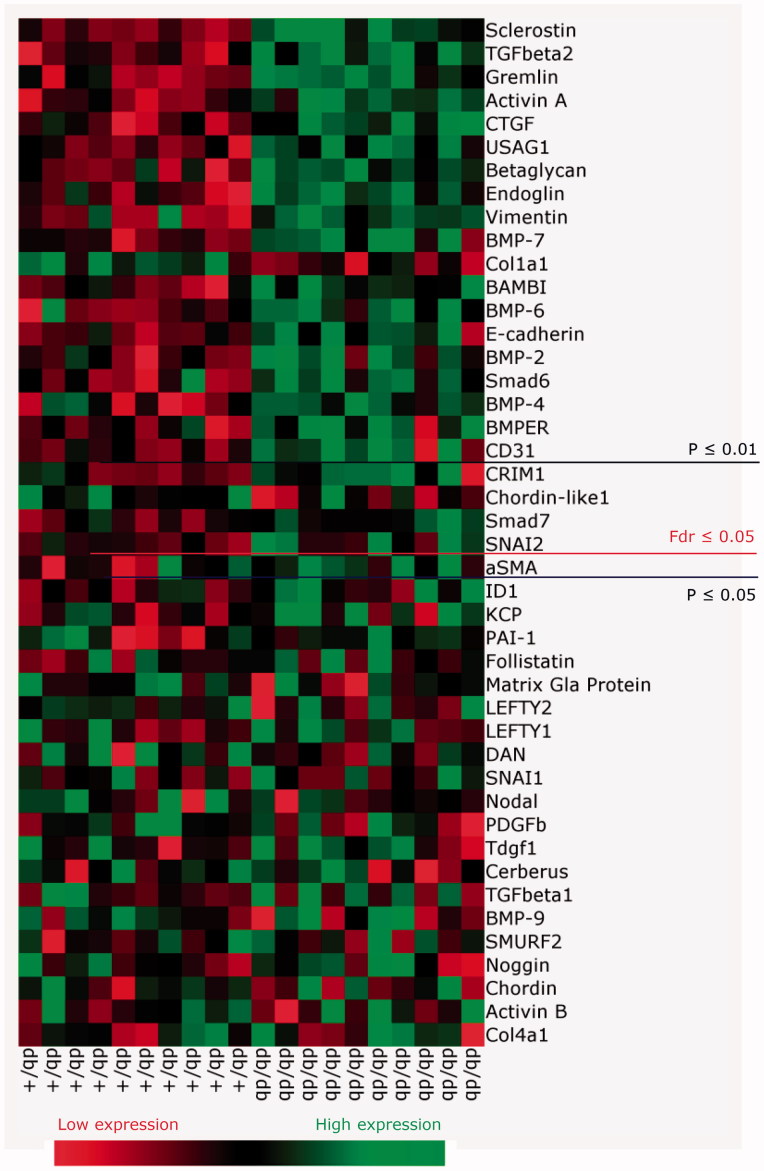
Heat map of expression of genes in the TGF-β family regulated in db/db animals vs. db/+ controls at 15 weeks of age. Values correspond to deltaCT values and in the online version of the manuscript the color coding corresponds to the relative expression in db/+ and db/db. High gene expression (a low deltaCT) is shown in green whereas low gene expression (a high deltaCT) is shown in red. Genes are ordered based on *p* values. The false discovery rate (Fdr) was set at .05 corresponding to a *p* values of .024.

**Figure 6.**
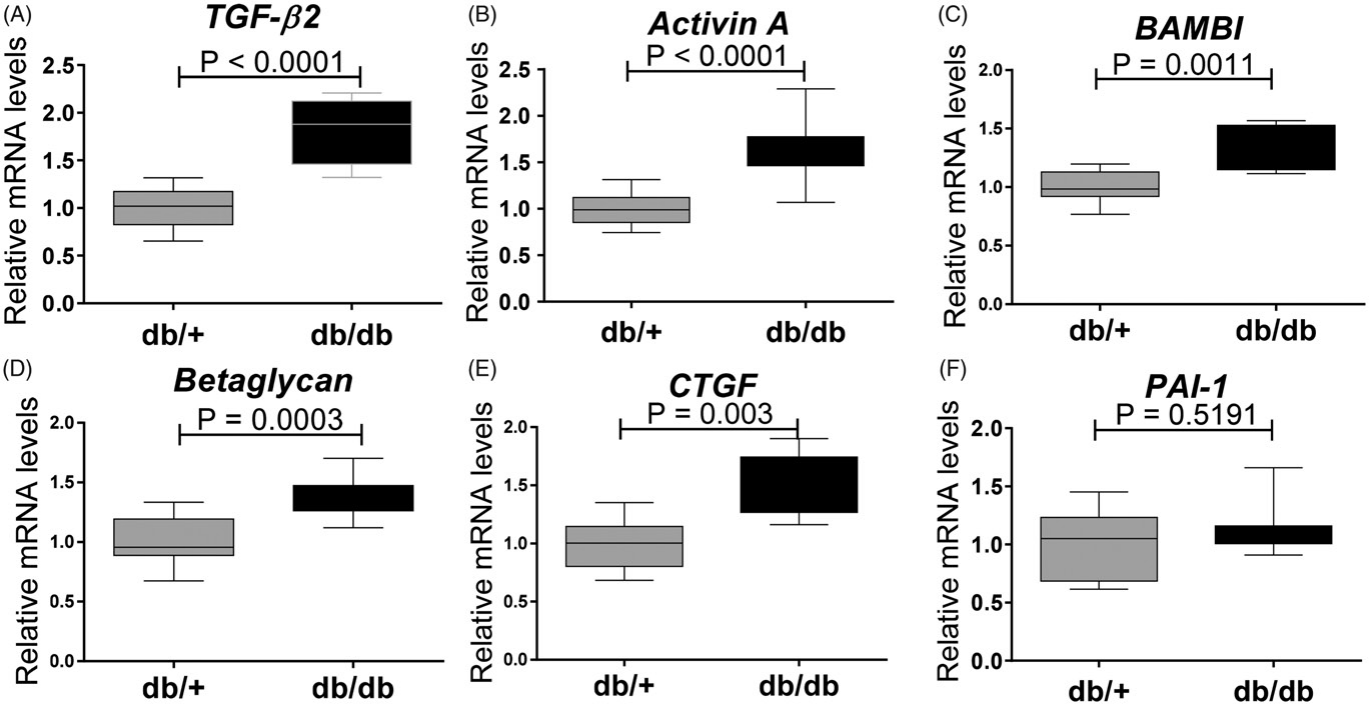
Expression of genes encoding modifiers of pSmad signaling in 15 weeks old db/db mice. (A) *TGF-β2*; (B) *Activin A*; (C) *BAMBI*; (D) *Betaglycan*; (E) *CTGF*; and (F) *PAI-1*. Values are relative mRNA levels corrected to housekeeping genes and displayed as boxes with whiskers showing minimum to maximum for a group of 10 mice.

RNA was extracted from whole kidneys and a heat-map displaying real time qPCR of TGF-β family genes for db/db and db/+ mice was generated. Based on 44 genes evaluated, 23 genes were modulated significantly (Figure 5). db/db kidneys showed significant up-regulation of genes associated with the TGF-β/Activin pathway such as *TGF-β2* (*p* < .0001) (Figure 6(A)), *Activin A* (*p* < .0001) (Figure 6(B)), and co-receptors of ligands such as *BAMBI* (Figure 6(C)) and *Betaglycan*, an accessory receptor for TGF-β (Figure 6(D)). The fibrotic TGF-β target gene connective tissue growth factor (*CTGF)* was also up-regulated in the diabetic mice (Figure 6(E)), but interestingly plasminogen activator inhibitor-1 (*PAI-1)* was not (Figure 6(F)). In contrast, *collagen1a1* was significantly down-regulated (Figure 5).

**Figure 7.**
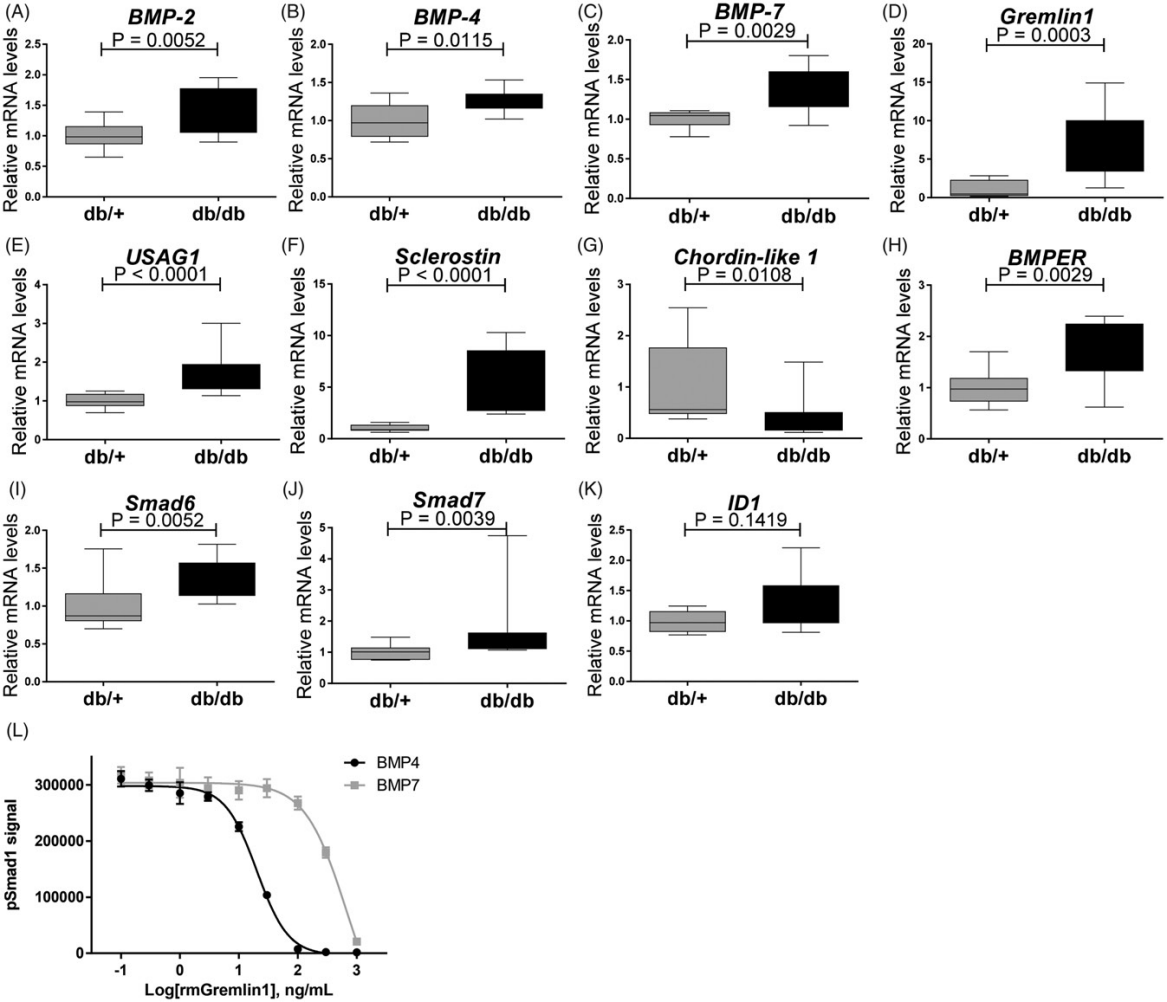
Expression genes encoding modifiers of pSmad signaling in 15 weeks old db/db mice. (A) *BMP-2*; (B) *BMP-4*; (C) *BMP-7*; (D) *Gremlin1*; (E) *USAG-1*; (F) *Sclerostin*; (G) *Chordin-like 1*; (H) *BMPER*; (I) *Smad6*; (J) *Smad7*; and (K) *ID-1*. Values are relative mRNA levels corrected to housekeeping genes and shown as box with whiskers minimum to maximum for a group of 10 mice. (L) Recombinant mouse Gremlin1 dose-dependent inhibition of BMP4 (4 ng/ml) and BMP7 (150 ng/ml) induced pSmad1 activity, quantified by AlhaLISA Surefire assay showing means ± SEM of *n* = 4.

Also BMP subfamily genes normally associated with activity of pSmad1/5/8 pathway were up-regulated (Figure 7). BMP ligands such as *BMP-2* (*p* = .0052) (Figure 7(A)), *BMP-4* (*p* = .0115) (Figure 7(B)), *BMP-6* (*p* = .0052) (Figure 5) and *BMP-7* (*p* = .0029) (Figure 7(C)) were up-regulated in the kidneys of diabetic db/db mice. BMP antagonists such as *USAG1* (*p* = .0001) (Figure 7(E)), *Sclerostin* (*p* = .0001) (Figure 7(F)), *Gremlin1* (*p* = .0003) (Figure 7(D)) and *BMPER* (*p* = .0029) (Figure 7(H)) were also strongly up-regulated, suggesting a dampening effect on overall BMP induced pSmad1/5/8 in the diabetic mice. *Chordin-like 1* was decreased in the db/db animals (Figure 7(G)). The inhibitory Smad proteins *Smad6* and *Smad7* (Figure 7(I,J)) were up-regulated in the db/db mice correlating with lack of regulation of BMP target gene expression such as *ID1* (Figure 7(K)). The increased expression of BMP antagonists, which are frequently induced by BMPs, may indicate the occurrence of a negative feedback loop.

Taken together, although the db/db mice showed up-regulation of the *TGF-β2* and *Activin A* ligands that might contribute to the induction of the target gene *CTGF*, this did not lead to up-regulation of other classical TGF-β regulated genes such as *PAI-1* and *collagen1a1*. Furthermore, the diabetic db/db kidney showed significant up-regulation of several BMP antagonists that might counteract the up-regulation of BMP target genes despite high ligand expression.

Since *Gremlin1* was up-regulated in diabetic db/db mice (Figure 7(D)), we hypothesized that the increased level of Gremlin1 within the diabetic animals could cause a dampening of pSmad1 signaling. Mouse PTEC cells, TCMK1, were stimulated with mouse BMP-4 and BMP-7 in the presence of mouse recombinant Gremlin1 protein to test this hypothesis. Here it was shown that Gremlin1 dose-dependently inhibits both BMP-4 and BMP-7 induced pSmad1, with the most potent effect on BMP-4 (Figure 7(L)).

## Discussion

In renal fibrosis, TGF-β family members and downstream Smad-dependent and independent pathways have been suggested to play a major role in tissue remodeling. In this study, the human DN kidneys showed up-regulated pSmad2 staining in tubular structures compared to healthy kidneys. The diabetic tissue showed an overall increased cytoplasmic expression and a small increase in pSmad2^+^ cells within the parietal cells of the Bowman’s capsule. Our results are important since clinical DN is characterized by a fibrotic stiffening of the epithelial compartment, possibly due to dysregulated TGF-β family activity and subsequent matrix deposition and EMT [36]. Hence the results obtained herein with the modulated expression patterns of pSmad2 might suggest direct participation of the TGF-β-family members in T2D-related DN disease progression.

We observed that human T2D kidneys were very fibrotic, showing dense and enlarged glomeruli. The ‘type 2-like’ db/db mouse model also showed enlarged glomeruli and dilated distal tubuli, but did not display markers of fibrosis. Enlarged glomeruli are possibly a consequence of hyperfiltration and hypertrophy whereas dilated tubuli may be a downstream characteristic of increased albumin filtration. These results support and extend a study by Ninichuk et al. [37] which reported the poor translation of the histological findings of tubular atrophy and interstitial fibrosis in db/db mice compared to humans. Recent reports show that the nodular sclerotic lesions in glomeruli are only observed in specific mouse strains with T2D that are lacking both the leptin gene and the leptin receptor [38,39]. The db/db mice did, however, show signs of damage by albuminuria, something others have described manifesting as late as 23–34 weeks of age [26,40,41]. Many experimental animal models of DN, including the db/db, only develop characteristics of earlier stages of DN due to the fact that their albumin filtration is increased without a subsequent decline in Glomerular Filtration Rate (GFR). We have previously confirmed this in house at our facility by conducting extensive measurement of both serum and urine inulin and creatinine without finding any decline in GFR for the time span tested in this study. Hence, differences in disease manifestation between mouse models and patients can be explained by a variety of different factors, where a very different time course of disease progression seems to complicate translation and should be addressed in preclinical development of therapeutic targets.

The elevated levels of pSmad2 observed in disease of the human kidney led us to study the translation to the experimental model of T2D-related DN, the db/db mouse. Surprisingly, db/db mice showed an opposite pSmad2 staining signature compared to what was observed in human T2D DN. Diabetic mouse kidneys showed significantly decreased pSmad2 staining within the PTECs. Not only did our results reveal a poor translation from the diabetic patient to the pre-clinical model with regards to molecular pSmad2 signature expression, but also the only minimal evidence of fibrotic changes found in the mice. It has been suggested that Smad2 signaling is anti-fibrotic and renoprotective, while it is Smad3 that is the pro-fibrotic factor [42,43]. Results on Smad staining within the field of kidney fibrosis are contradictory, which we suggest can be due to different mouse strains and the use of different anti-Smad antibodies directed against either phospho-Smad or Smad showing different affinity and degree of cross-reactivity. We see the advantage of adding our findings with an actual activated Smad staining with a validated antibody to the field that has no cross-reaction to other phosphorylated Smads on immunoprecipitation (see the ‘Methods’ section). We used pSmad2 staining to evaluate overall TGF-β activity in kidneys from patients and mouse model and downstream profibrotic gene expression to evaluate effect on fibrotic pathways in the mouse model.

T2D patients show up-regulated *PAI-1* gene levels [44], whereas we did not observe expression changes in the db/db mice. This is despite the fact that ligands such as *Activin A* and *TGF-β2,* both thought to mediate pSmad2/3 signaling, were up-regulated in the diabetic db/db kidney. In another model of fibrotic kidney disease, unilateral ureteral obstruction, Activin A has previously been reported up-regulated locally in tubular cells were it was speculated to act as a paracrine factor activating renal interstitial fibroblasts in the kidney to produce collagen and α-SMA [45]. While *TGF-β2* was up-regulated in db/db tissue on gene level, *TGF-β1* was surprisingly not up-regulated. There is a great redundancy between the TGF-β-ligands as all TGF-β isoforms has been reported to induce fibrosis [46]. The role of the different TGF-β isoforms in T2D associated DN is still not fully understood and many studies report, unlike our db/db data, *TGF-β1* to be up-regulated in the glomerulus and in the cortical tubules [47,48].

Despite ligands favoring activation of the Smad2/3 pathway being up-regulated, the analysis of the TGF-β family on a gene level showed strong up-regulation of antagonists of the pSmad2 pathway: *BAMBI*, a TGF-β family antagonist was up-regulated in diabetic mice vs. controls. Furthermore, *Betaglycan,* a co-receptor of all TGF-β isoforms, but especially important for TGF-β2 and TGFβRII assembly, was also up-regulated in db/db mice, *Betaglycan* was also up-regulated in mice 15 weeks of age. However, in some studies Betaglycan has an inhibitory effect to TGF-β signaling which might contribute to the lack of pSmad2 evidenced in db/db [49,50]. Studies in db/db have shown that soluble Betaglycan reduce the expression of all TGF-β isoforms and additionally reduce the kidney levels of collagen IV and fibronectin [51]. Hence, even though *CTGF*, a downstream target of TGF-β, was up-regulated, the fibrotic *PAI-1* was not up-regulated, correlating with absent regulation of collagens and extracellular matrix found in the diabetic mice (neither on gene level or protein level with histology). This could suggest a limited role of TGF-β induced fibrotic events in the db/db model of DN.

Translation between human disease and pre-clinical models has been a challenge for many diseases and particularly for DN [34]. Most focus concerning the role of TGF-β family in DN has been on TGF-β1 and BMP-7, and to our knowledge, limited literature exists on the remaining part of the BMPs within DN. The level of pSmad1 was not examined in the human samples of this study, but other groups have reported contradictory results within this field. Turk et al. [52] has reported less pSmad1/5/8 in progressed human DN, while Abe et al. [53] has reported up-regulated Smad1 in advanced DN. The diabetic mice of this current study did not show any difference of pSmad1/5/8 protein expression even though several BMP ligands were up-regulated. In contrast, Smad1 expression was shown to correlate with the severity of mesangial matrix expansion in STZ-treated Sprague–Dawley rats [54], and similar to our study, Matsubara et al. [55] also found several BMP ligands up-regulated in STZ mice on a C57BL/6 J background. It is possible that we looked at a too early time point in db/db renal disease progression or that the db/db model simply does not show the same pSmad patterns as the STZ model. Alternatively, the unchanged level of pSmad1 may be explained by up-regulation of BMP antagonists such as *Gremlin1, Sclerostin, USAG1* and *BMPER,* which were all induced in the kidneys of db/db mice. The role of these antagonists is to control BMP signaling, frequently as part of a negative feedback loop. In line with this, the downstream BMP target *ID1* was not regulated.

Gremlin1 is an antagonist of BMP-2, BMP-4 and BMP7, which heterodimerizes with the ligands and thereby prevents them from binding to their receptors [56]. Patients with severe DN show high expression of Gremlin1 in tubular epithelial cells [57] where expression correlates with tubulointerstitial damage and elevated serum creatinine levels [58]. Interestingly, transgenic STZ mouse expressing human Gremlin1 in PTECs show greater glomerular and tubulointerstitial injury [59] and heterozygous Gremlin1 knockout mice ameliorates renal damage [59]. We showed that recombinant mouse Gremlin1 was able to inhibit pSmad1 signal in BMP-4 and BMP-7 stimulated mouse PTECs, which therefore, could present a mechanistic explanation for the lack of increased pSmad1 despite increased BMP-2, -4 and -7.

## Conclusions

Fibrotic disease represents one of the largest groups of disorders for which there is no effective therapy and thus proving a major unmet medical need. T2D associated DN has not been given the same attention as T1D due to the complexity of disease causality. This study provides new insights into DN and the regulation of TGF-β family members in a classical pre-clinical T2D model. In summary, translation of the development of fibrosis and DN between T2D patients and the ‘type-2’-like experimental pre-clinical model, db/db mice is poor on a macroscopic as well as on a molecular level, emphasizing that the choice of preclinical model should be considered carefully. The current study provides important new information on the role of TGF-β family regulation and highlights that monitoring signaling events and TGF-β family expression patterns could be useful as biomarkers or as pharmaceutical target in DN.
